# Compressive loading of the murine tibia reveals site-specific micro-scale differences in adaptation and maturation rates of bone

**DOI:** 10.1007/s00198-016-3846-6

**Published:** 2016-12-05

**Authors:** I. Bergström, J. G. Kerns, A. E. Törnqvist, C. Perdikouri, N. Mathavan, A. Koskela, H. B. Henriksson, J. Tuukkanen, G. Andersson, H. Isaksson, A. E. Goodship, S. H. Windahl

**Affiliations:** 1 Department of Endocrinology, Metabolism and Diabetes, Karolinska University Hospital, Karolinska Institutet, Stockholm, Sweden; 2 UCL Institute of Orthopedics and Musculoskeletal Science, Royal National Orthopedic Hospital, London, UK; 3 Lancaster Medical School, Faculty of Health and Medicine, Lancaster University, Lancaster, LA1 4YG UK; 4 Rheumatology and Bone Diseases Unit, Centre for Genomic and Experimental Medicine, MRC Institute of Genetics and Molecular Medicine, Western General Hospital, University of Edinburgh, Edinburgh, EH4 2XU UK; 5 Department of Biomedical Engineering and Department of Orthopedics, Lund University, Lund, Sweden; 6 Institute of Cancer and Translational Medicine, Department of Anatomy and Cell Biology, MRC Oulu, University of Oulu, Oulu, Finland; 7 Department of Orthopedics, Institute of Clinical Sciences, Sahlgrenska Academy, University of Gothenburg, Gothenburg, Sweden; 8 Department of Orthopedics, Sahlgrenska University Hospital, Gothenburg, Sweden; 9 Department of Laboratory Medicine, Division of Pathology, Karolinska University Hospital, Karolinska Institutet, Huddinge, Stockholm, Sweden; 10 Centre for Comparative and Clinical Anatomy, School of Veterinary Science, University of Bristol, Bristol, UK; 11 Centre for Bone and Arthritis Research, Institute of Medicine, Sahlgrenska Academy, University of Gothenburg, Gothenburg, Sweden

**Keywords:** Loading, Raman spectroscopy, RPI, SAXS

## Abstract

**Summary:**

Loading increases bone mass and strength in a site-specific manner; however, possible effects of loading on bone matrix composition have not been evaluated. Site-specific structural and material properties of mouse bone were analyzed on the macro- and micro/molecular scale in the presence and absence of axial loading. The response of bone to load is heterogeneous, adapting at molecular, micro-, and macro-levels.

**Introduction:**

Osteoporosis is a degenerative disease resulting in reduced bone mineral density, structure, and strength. The overall aim was to explore the hypothesis that changes in loading environment result in site-specific adaptations at molecular/micro- and macro-scale in mouse bone.

**Methods:**

Right tibiae of adult mice were subjected to well-defined cyclic axial loading for 2 weeks; left tibiae were used as physiologically loaded controls. The bones were analyzed with μCT (structure), reference point indentation (material properties), Raman spectroscopy (chemical), and small-angle X-ray scattering (mineral crystallization and structure).

**Results:**

The cranial and caudal sites of tibiae are structurally and biochemically different within control bones. In response to loading, cranial and caudal sites increase in cortical thickness with reduced mineralization (−14 and −3%, *p* < 0.01, respectively) and crystallinity (−1.4 and −0.3%, *p* < 0.05, respectively). Along the length of the loaded bones, collagen content becomes more heterogeneous on the caudal site and the mineral/collagen increases distally at both sites.

**Conclusion:**

Bone structure and composition are heterogeneous, finely tuned, adaptive, and site-specifically responsive at the micro-scale to maintain optimal function. Manipulation of this heterogeneity may affect bone strength, relative to specific applied loads.

**Electronic supplementary material:**

The online version of this article (doi:10.1007/s00198-016-3846-6) contains supplementary material, which is available to authorized users.

## Introduction

Bone mineral density is a major parameter influencing bone material properties, stiffness, and elastic modulus. Bone “quality” includes all factors contributing to the strength of the bone and is characterized by a combination of the geometry, micro-architecture (three-dimensional organization), bone turnover, micro-damage, and bone matrix chemistry (mineral and collagen content) [1, 2]. The bone matrix consists of both organic (~35%) and inorganic (~65%) components [3]. The organic component of bone is composed of up to 90% type I collagen and, together with the mineral component, governs the biomechanical properties, strength, toughness, and functional integrity of the tissue [4, 5]. Collagen has a mechanical function in all connective tissues contributing to tensile strength and structural integrity. Several studies indicate that the collagen component has a substantial role in the toughness of bone (capacity to absorb energy), while the mineral content mainly determines the stiffness of bone [1]. Thus, bone strength is affected by both the relative amounts and the geometric organization of the bone matrix but also the specific chemistry of the organic and inorganic components.

Long bones in rodents are heterogeneous in that bone formation and resorption occur at the bone surfaces while the intracortical area seems to remain constant, because there is no osteonal remodeling [6, 7]. This heterogeneity is reflected in the distribution of bone mineral density, mineral crystal composition and direction, and collagen maturity [7–9]. Thus, the material properties depend on the relative composition of the bone matrix components, and the interaction between them, which subsequently affect the structural properties. In humans, bone turnover occurs primarily through the process of secondary osteonal remodeling. This leads to regions with bone of different age in relation to the age of the individual. The heterogeneity of bone also facilitates redistribution of strain from the surface, thus avoiding micro-cracks and improving mechanical properties [10]. Renders et al. have suggested that the distribution of stress and strain is favored by the mineral heterogeneity in human intratrabecular (within individual trabeculae at the tissue level) bone [11]. This influences elasticity as well as the fracture risk. Spatial heterogeneity is decreased in bone exposed to bisphosphonates in both animal and human studies [12–14], and heterogeneity in the degree of mineralization is decreased in postmenopausal women with hip fractures treated with bisphosphonates [13]. Although bones exposed to bisphosphonate treatment do respond to strain from applied load [15], it is not known if the bisphosphonate treatment alters the heterogenic pattern of the anabolic response of bone to the applied strains and, if so, would interfere with an appropriate anabolic response of bone to loading.

Imposed loading of bone with magnitudes and direction that differ to physiological loading will induce modeling, which results in bone formation on periosteal and endosteal surfaces [16, 17]. Although the exact impact of changes in bone heterogeneity on mechanical properties has not been analyzed directly, it is important to consider bone tissue heterogeneity as a bone quality factor. Loading of mouse bone has beneficial effects on the mechanical properties [18]. Especially in young growing mice, physiological loading improved both the collagenous and mineral mechanical properties of bone, while the mineralized bone mechanical properties were mostly improved in elderly mice [18]. Other studies using a controlled imposed loading regime indicate that the effect on the bone composition may be age-dependent, with enhanced collagen maturity in elderly mice [19]. Thus, the literature is generally conclusive of the beneficial response of loading, but the extent of the adaptive response is highly sensitive to the type and pattern of loading, age of the animal, etc.

Vibrational spectroscopic techniques, such as infrared or Raman spectroscopy, have been used to study the chemical composition of the bone matrix in healthy and diseased bone [20, 21]. Development of Raman spectroscopy in living subjects has allowed for subsurface spectra to be acquired; i.e., it is now possible to interrogate bone matrix chemistry through the skin [22]. A recent study investigated changes in Raman spectra due to different mineralization levels of various animal bones evolved for different physiological requirements. Discernible differences between the highly mineralized brittle ear bone and less mineralized tough antlers were found, specifically in the case of the red deer [23]. Recent data also indicated variations in matrix chemistry along the length of a human tibia, showing that bone adaptation is complex with not only structural optimization on the micro-scale but also local material adaption on the molecular scale [20]. Whether these local differences are due to different remodeling rates along the bone length, where the newer bone would be less mature, or if it is due to differences in strain levels resulting in site-specific differences in local adaptation and therefore more persistent differences in bone quality and strength, is unknown.

The overall aim was to explore the hypothesis that changes in loading environment result in site-specific adaptations at molecular/micro- and macro-scale in mouse bone.

## Materials and methods

### Animals

Three-month-old mature C57BL/6 female mice (Charles River Laboratories, Germany) were housed in a standard animal facility under controlled temperature (22 °C) and photoperiod (12-h light, 12-h dark). They were fed a pellet diet and given access to drinking water ad libitum.

### Ex vivo mechanical strain measurement during dynamic axial loading of tibia

The magnitude of axial mechanical strain applied to the tibia during loading was established ex vivo as previously described [17]. Five 16-week-old female C57BL/6 mice were killed and directly used for the ex vivo strain gauging measurements. An incision was made in the skin of the tibiae, soft tissue was removed, and a single element strain gauge was attached (EA-06-015DJ-120, Vishay Measurement Group, PA, USA), in longitudinal alignment with the medial (tensile) aspect of the tibia at 37% of its length from the proximal end. Previous studies have shown that this region corresponds to the site of greatest osteogenic response to axial loading, and the 37% site is often used as a representative part of this region [16, 24, 25]. Strains were measured across a range of peak compressive loads between 7 and 17 N (Fig. S1). These peak loads were applied with the same 3100 ElectroForce® Test Instrument (Bose Corporation, MN, USA) with the same holding cups that were used for in vivo loading. From the data, a specific peak load (13 N) corresponding to 2700 ± 600 με was used in the loading experiment. The load and strain used were in line with previous studies by our and other groups and are not known to be associated with micro-damage in the bones [16].

### In vivo loading of the tibia

While under inhalation anesthesia with isoflurane (Forene; Abbot Scandinavia, Solna, Sweden), the right tibiae of seven 16-week-old female C57BL/6 mice were axially loaded as previously described [16, 26]. The right tibiae were loaded on three alternate days per week for 2 weeks, in total six loading bouts. The loading was applied with 40 cycles/day, at 1 Hz, using a trapezoid waveform and a 10 s rest between cycles. The loads were applied using a 3100 ElectroForce® Test Instrument (Bose Corporation, MN, USA). The left tibia was used as a physiologically loaded control (hereafter referred to as “non-loaded”) to allow like-for-like comparisons for the effects of loading on bone modeling. The use of the contra-lateral limb as a control using this protocol has been validated previously [16, 25]. All mice were healthy and allowed normal cage activity in between loading sessions. The mice were euthanized and their tibiae were dissected, wrapped in saline soaked gauze, and kept frozen at −20 °C until analysis.

### Raman spectroscopy

Five Raman spectra were acquired from 37% of the length of the tibia, as measured from the proximal end, of both cranial and caudal sites (Fig. S2A, *N* = 7), using an inVia Raman micro-spectrometer (Renishaw plc, Gloucestershire, UK). The bones were kept hydrated with PBS-soaked gauze during scanning, with only the area scanned exposed at a time. The instrument was equipped with an 830 nm 300 mW laser, spectra were acquired for 10 s and 60 accumulations, and the spot size of the laser was 2 × 2 μm, with power over this area ~2 mW. For four bones (2× non-loaded; 2× loaded), six additional spectra were acquired along the length, from proximal to distal, at the cranial region, using the same settings as above. Ratios were compared along the length of the bone from the distal, proximal, and middle regions. All spectra were baseline corrected using an in-house written script in Matlab (MATLAB 2012a, MathWorks, Inc., USA) and normalized to the phosphate peak (960 cm^−1^). Ratios were calculated by comparing the heights of the bands: phosphate, amide I (1660 cm^−1^), carbonate (1070 cm^−1^), proline (920 cm^−1^), and hydroxyproline (870 cm^−1^). The ratio bioapatite/collagen is a good measure of total collagen content and was determined by dividing the height of the phosphate peak by the combined height of the proline and hydroxyproline peak heights [27]. Determining crystallinity by calculating the inverse of the width of the hydroxyapatite (phosphate) peak at half the height has been previously proven as suitable to assess mineral crystal maturity [28, 29]. Larger crystal minerals are more mature, resulting in a narrow peak, and smaller mineral crystals are less mature, resulting in a wider peak [29] .

### Micro-computed tomography

The tibiae (*N* = 7) were fixed and stored in 70% ethanol before micro-computed tomography (μCT) analysis. High-resolution μCT analysis was performed on the diaphyseal region of the tibia (1172 model, Bruker microCT, Aartselaar, Belgium). The tibiae were imaged with an X-ray tube voltage of 50 kV and current of 201 μA, with a 0.5 mm aluminum filter. The scanning angular rotation was 180° and the angular increment 0.70°. The voxel size was 4.48 μm. Images were reconstructed using NRecon (version 1.6.9). The equipment was calibrated with standard phantoms provided by the manufacturer (BMD calibration phantoms, 0.25 and 0.75 g/cm^3^, Bruker microCT, Aartselaar, Belgium). All images were analyzed using CTAn (version 1.13.10.1+), giving the cortical bone area (B.Ar) and cortical thickness (Ct.Th). The average cortical thickness (Ct.Th) was calculated using an automatic internal algorithm included in the CTAn software. The site-specific CT.Th was measured manually; an average from 10 measurements per bone was calculated. Analysis of cortical bone started at a distance of 5.36 mm distal to the growth plate and extended longitudinally 134.5 μm in the distal direction, corresponding approximately to 37% of the length of the tibia from the proximal end. A square cuboid of 100 μm × 100 μm × 134.5 μm was used for the caudal region and a diameter for a cylinder of 500 μm and length of 134.5 μm for the caudal region. Different shapes (a square cuboid at the cranial site, and circular shape at the caudal site) were used to accommodate the differences in shape of the bone in these two regions (Fig. S2C).

### Small-angle X-ray scattering

The same tibiae as described above were imbedded in plastic. Sections (70 μm) were sawed transversely from the embedded tibia from four mice per group at approximately 37% proximal of the total length of the tibia (non-loaded and loaded). SAXS measurements were conducted at the I911-4 SAXS beamline at the 1.5 GeV ring (MAX II) of the MAX IV Laboratory (Lund University, Lund, Sweden) [30]. The wavelength was 0.91 Å, and the size of the synchrotron X-ray beam at the sample region was approximately 0.1 × 0.1 mm^2^. The Pilatus 1 M detector was placed at 1900 mm behind the sample, and the exposure time to collect each SAXS pattern was 5 s. The *q*-range measured was 0.01–0.30 Å^−1^. A motorized x-y scanning stage was used to map the sample with a step size of 0.1 mm in both directions. A ~1 × 1 mm area at both regions of each sample was measured (Fig. S2B).

The data analysis has been described in detail previously [30]. In brief, from the 2D SAXS intensity pattern, the mineral plate thickness, predominant orientation, and degree of orientation were determined for each measurement point [31–33]. From this pattern, the mineral plate thickness was evaluated following the approach suggested by Bünger et al. [31]. It is based on a curve fitting where the mineral crystals are assumed to be plates with a finite thickness, *T*, in one dimension, and infinite size in the other two dimensions. The predominant orientation and the degree of orientation were determined such that the first is where the intensity reaches its maximum, and the second is a calculated value between 0 and 1, where 0 corresponds to no predominant orientation within the plane of the section, and 1 that all mineral crystals are aligned perfectly in the same direction [9, 30, 34]. All analyses were done using custom-made Matlab scripts (MATLAB R2011b, MathWorks, Inc., Natick, MA, USA).

### Reference point indentation

Reference point indentation (RPI) (BioDent Hfc, ActiveLife Scientific, CA, USA) was performed at 37% of the length of the tibia from the proximal end of each region (cranial and caudal) and along the length of each bone at both regions (*N* = 7, Fig. S2A); due to the size of the bones, a maximum of seven indentations were made along the length of each bone, and for each region. Using a BP2 test probe, a reference force of ~1300 g was applied, followed by a force of 2 N for 10 cycles at 2 Hz; samples were kept hydrated throughout testing. The total indentation distance relates to fracture risk [35], and the unloading slope correlates to the stiffness of the material. Data for total indentation distance and unloading slope were averaged and standard deviations calculated. PMMA was used as a calibration specimen, and repeated measurements had standard deviations of <0.5. For the total indentation distance, the mean and confidence interval at 95% associated with the mean estimate are reported. Immediately after the RPI, the tibiae were fixed in 4% paraformaldehyde for 2 days and then stored in 70% ethanol. Due to the small nature of the mouse bones, RPI data were collected along the length of each bone and analyzed proximal to distal, as well as comparing loaded to non-loaded, of the cranial and caudal regions respectively (Fig. S2A).

### Statistics

The mean and confidence interval at 95% associated with the mean estimate were calculated and reported. Student’s *t* test, one-way ANOVA, 2 × 2 ANOVA, or mixed model followed by the Bonferroni post hoc test was performed as appropriate. On the spectroscopic data, multivariate analysis was performed (principal component analysis in combination with linear discriminant analysis, PCA-LDA). LDA was employed on this data to force separation based on assigned classes (e.g., loaded vs. non-loaded). 1D scatter and loading (pseudo-spectra visualizing any variance) plots were produced to enable identification of the spread of the data, and the cause of the spread, respectively. A *p* value ≤0.05 was considered significant.

## Results

### Caudal vs. cranial response to load in the proximal region

#### Bone matrix chemistry

**Fig. 1:**
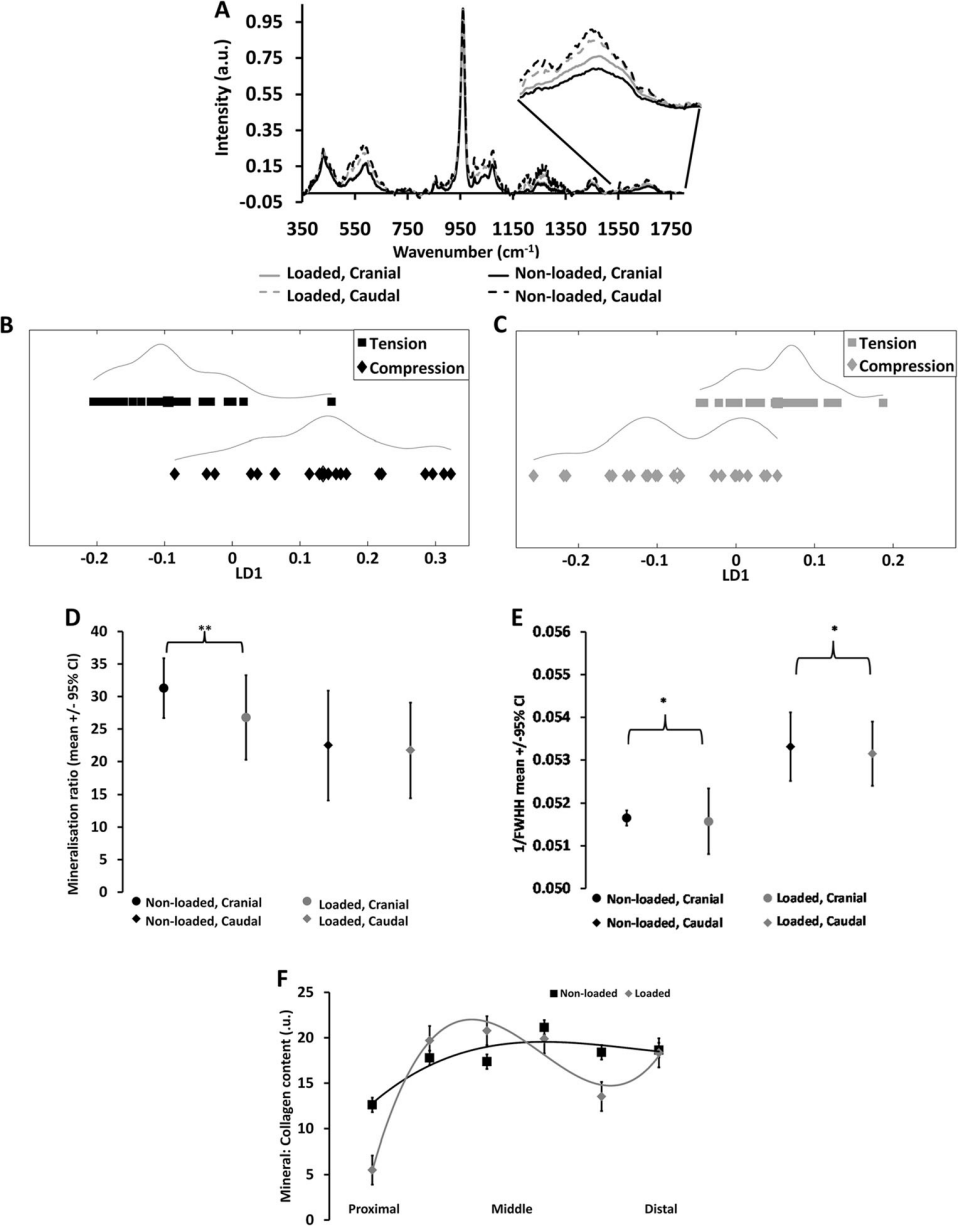
Site-specific differences in bone chemistry. **a** The average Raman spectrum for each region and each cohort (loaded or non-loaded). The cranial region is *black* and the caudal region is *gray*. A *broken line* is used for non-loaded, and a *continuous line* for loaded. The *inset* is a close-up of the amide I peak, where a lower peak indicates a lower mineralization ratio. **b** The spread of spectral data, analyzed by PCA-LDA, across the two regions for the non-loaded bones. **c** The spread of spectral data, analyzed by PCA-LDA, across the two regions for the loaded bones. **d** The average mineralization ratios +/− 95% confidence interval for each cohort of bones and at each region. **e** The level of crystal maturity/size as determined by the inverse of the full width at half height of the phosphate peak. *p < 0.05 and **p < 0.01. **f** The collagen content variation along the length of the bones, calculated from the Raman spectra; a third-order polynomial has been fitted to each set of data. Data presented along the length are spectra acquired along the caudal side from four bones (please see further details in the “Methods” section); all other spectra displayed were acquired from a position 37% from the proximal end of the bones and from the caudal and cranial sides, labeled accordingly

We analyzed the cranial and caudal sides of the proximal part of the tibiae corresponding to approximately 37% of the total length from the knee where the main response to axial loading is seen. The average Raman spectrum for each cohort and region showed that the cranial site was more mineralized than the caudal site, both in the non-loaded and loaded bones (Fig. 1a). Analysis using PCA scatter plots shows that the spread of the data was different for the non-loaded (Fig. 1b) and loaded (Fig. 1c) bones across the regions. Specifically, differences between the non-loaded sites were due to phosphate peak. The distance between points is proportional to biochemical similarity in these plots. The non-loaded plot (Fig. 1b) shows that the cranial and caudal sites are biochemically different from each other at the phosphate peak. In addition, the caudal site is more heterogeneous than the cranial site. This also holds for the loaded plot (Fig. 1c), which reveals two subpopulations within the caudal site. Furthermore, differences between the loaded cranial and caudal sites were due to phosphate and carbonate.

Univariate analysis, using the phosphate bandwidth, was used to investigate the degree of mineralization and mineral crystal size at the different regions (Fig. 1d, e and Table 1). The cranial site of non-loaded bones displays a significantly higher mineralization ratio (*p* < 0.01), but smaller mineral crystal size than the caudal site. In response to load, the cranial site significantly decreases its mineralization ratio (*p* < 0.01) and mineral crystal size (*p* < 0.01), while the caudal site decreases only its mineral crystal size (*p* < 0.05), but not its mineralization ratio in response to load (Fig. 1d, e and Table 1).

**Table 1:** Mineralization, and crystal maturity changes in response to loading

	Tension		Compression	
	Non-loaded	Loaded	Non-loaded	Loaded
Mineralization	31.28 ± 2.33	26.77 ± 3.30*	22.5 ± 4.28	21.75 ± 3.74
Crystal maturity	0.052 ± 0.000	0.052 ± 0.001*	0.053 ± 0.001	0.053 ± 0.001*

Mineralization and crystal maturity/size in response to loading measured by Raman spectroscopy. Mean ± SEM are shown. Significance between non-loaded and loaded indicated at the latter with *, p<0.05

**Fig. 2:**
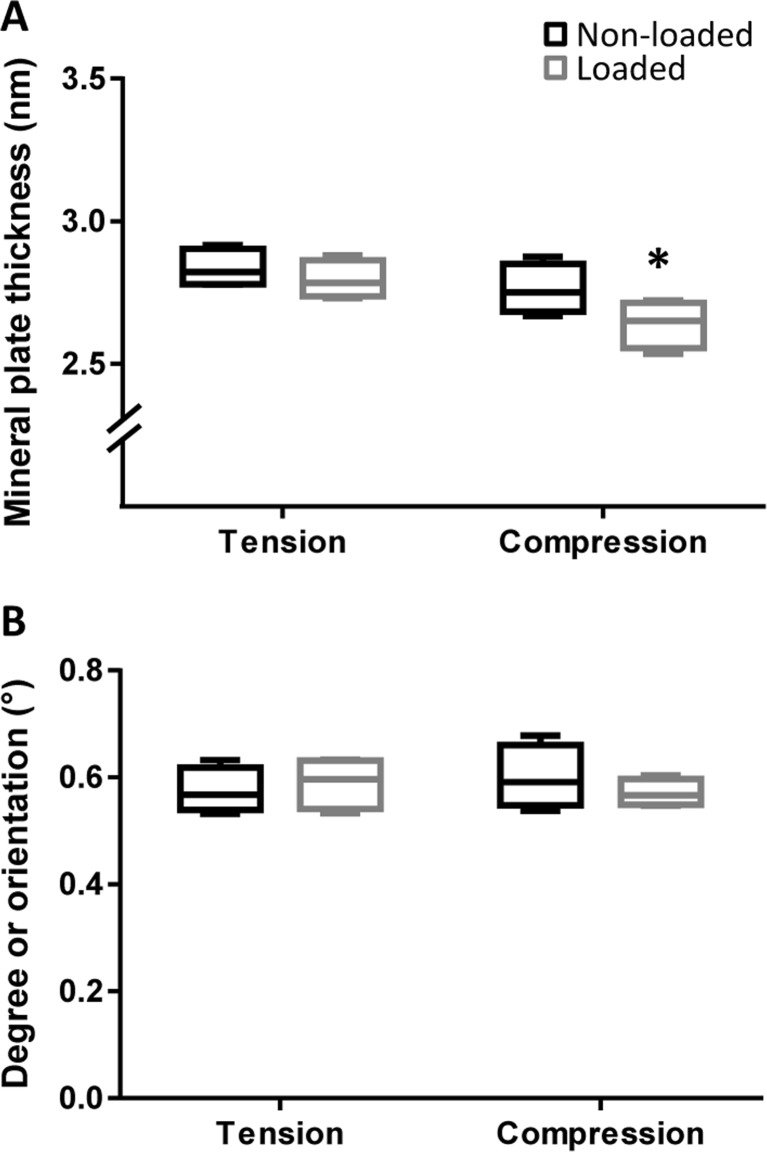
Loading decreases crystal plate thickness caudally SAXS analysis. **a** crystal plate thickness, **b** degree of mineral crystal orientation are shown as mean +/− 95% confidence interval. Statistics: mixed procedure in SPSS with post hoc Bonferroni correction, *p < 0.05 for non-loaded vs. loaded

Further analysis of the mineral crystals using SAXS did not detect any differences in the mineral plate thickness between the cranial and caudal regions in non-loaded bones (Fig. 2a). However, in the loaded bones, the mineral plate thickness was significantly reduced at the caudal site. The degree of orientation of the mineral crystals was similar in both the cranial and caudal sites and did not change in response to load (Fig. 2b).

#### Bone material properties

**Fig. 3:**
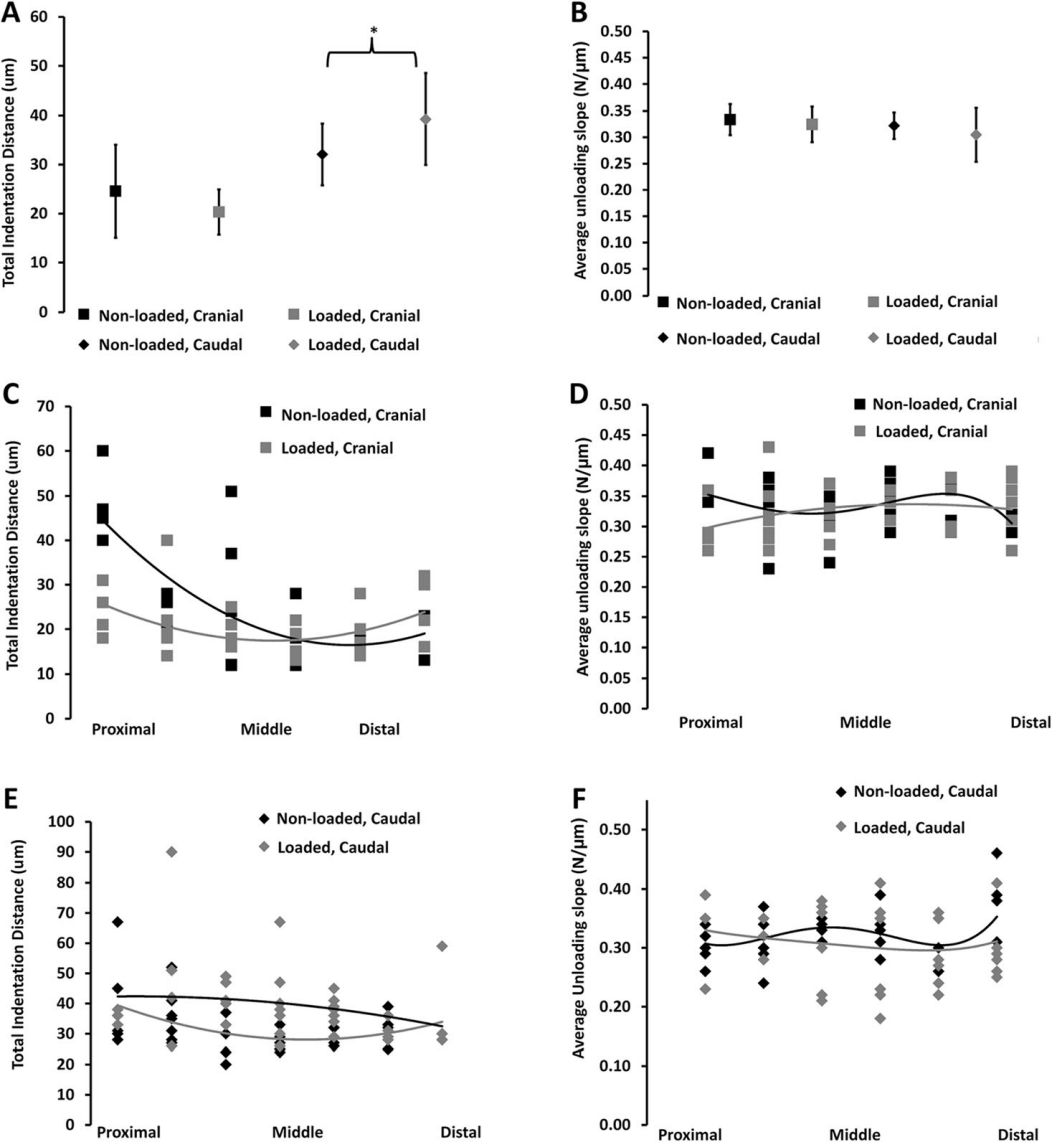
The material properties of the caudal region are less tough. **a**, **c**, **e** The total indentation distance. **b**, **d**, **f** The unloading slope (US) using RPI. **a**, **b** The average measure +/− 95% confidence interval across both regions for non-loaded vs. loaded. **c**, **d** The cranial region from proximal to distal along each bone for non-loaded vs. loaded. A third-order polynomial has been fitted to each set of data. **e**, **f** Caudal region from proximal to distal along each bone for non-loaded vs. loaded; a third-order polynomial has been fitted to each set of data

In the non-loaded control bones, the main difference between the energy dissipation was 44% higher at the caudal side than the cranial site (Table 2). However, for the loaded side, the most significant difference was in stiffness (*p* < 0.05, average loading slope), which was 12% higher at the cranial site than the caudal site, further supporting the findings described above. In response to load, the total indentation distance increased significantly in the caudal site, exposed mainly to compression loading (+21%, *p* < 0.05) (Fig. 3a), and the energy dissipation increased (+44%, *p* < 0.05, Table 2).

**Table 2:** RPI measurement, non-loaded vs. loaded for tension and compression

	Tension		Compression	
	Non-loaded	Loaded	Non-loaded	Loaded
1st-cycle indentation distance (ID 1st), μm	21.72 ± 2.22#	18.20 ± 1.04#	29.58 ± 1.32	36.33 ± 1.98*
1st-cycle unloading slope (US 1st), N/μm	0.34 ± 0.01	0.33 ± 0.01	0.33 ± 0.01	0.32 ± 0.01
1st-cycle creep indentation distance (TID 1st-L), μm	2.48 ± 0.27	2.11 ± 0.15#	3.03 ± 0.16	4.00 ± 0.38
Total indentation distance (TID 1st-L), μm	24.55 ± 2.37#	20.46 ± 1.06#	32.05 ± 1.37	39.22 ± 2.09*
Indentation distance increase (ID 1st-L), μm	4.79 ± 0.54	3.89 ± 0.29#	5.05 ± 0.32	6.22 ± 0.52
Avg creep indentation distance (Avg CID 1st-L), μm	1.00 ± 0.07	1.03 ± 0.03	1.03 ± 0.03	1.16 ± 0.06*
Avg energy dissipated (Avg ED 3rd-L), μJ	3.25 ± 0.29#	3.03 ± 0.36	2.49 ± 0.17	3.60 ± 0.20*
Avg unloading slope (Avg US 1st-L), N/μm	0.34 ± 0.01	0.32 ± 0.01	0.32 ± 0.01	0.31 ± 0.01
Avg loading slope (Avg LS 1st-L), N/μm	0.25 ± 0.01	0.25 ± 0.01#	0.26 ± 0.01	0.22 ± 0.01*

Mean +/− SEM for each RPI output measurement. Analyzed using ANOVA (SPSS): significant differences between tension (non-loaded vs. loaded) and compression (non-loaded vs. loaded) are denoted by *p < 0.05, and between non-loaded (tension vs. compression) and loaded (tension vs. compression) denoted by #*p* < 0.05

#### Bone structure

**Fig. 4:**
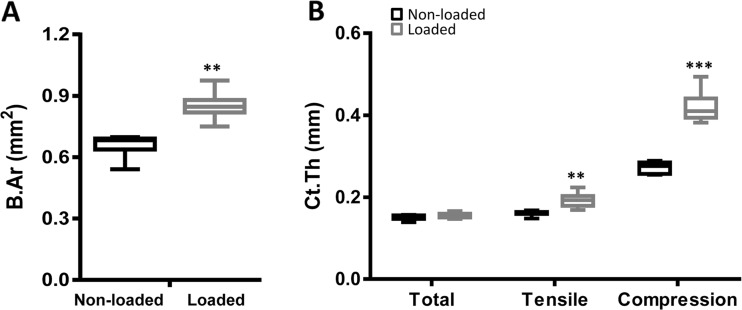
The caudal site is thicker and responds more to loading μCT analysis. **a** cortical area and **b** cortical thickness of loaded and non-loaded bones at the mid-diaphysis of the tibiae are shown as mean +/− 95% confidence interval. Cortical thickness is given as average for the whole bone, or specifically for the cranial, or caudal sites. Statistics: **a** Student’s *t* test, **p < 0.01 for non-loaded vs. loaded. **b** ANOVA in SPSS with Bonferroni post hoc comparison, **p < 0.01 and ***p < 0.001 for non-loaded vs. loaded

μCT was used to study alterations in overall cortical bone parameters, specifically in the regions representing the cranial and caudal areas (Fig. S2C, in non-loaded and axially loaded tibiae, respectively. Loading significantly increased the total cortical area (29%, *p* < 0.01; Fig. 4a), resulting in a significant increase in mean polar moment of inertia (an estimate of resistance to torsion) (+35%, *p* < 0.01, data not shown), showing that the loading protocol resulted in an overall anabolic response.

There were site-specific differences in cortical thickness within the non-loaded bones where the caudal side of the non-loaded bone displayed a significantly larger cortical thickness (Fig. 4b, +82%; *p* < 0.001), compared to the total cross-sectional thickness. In response to load, the total cross-sectional cortical thickness did not change significantly. However, site-specific changes were observed in response to load. On both the cranial and the caudal sites, there was a significant increase in cortical thickness (Fig. 4b, +20.8%; *p* < 0.001 and +53%; *p* < 0.0001, respectively).

### Along the length (caudal vs. cranial) in response to load

#### Bone matrix chemistry

Analyses along the length of the bones showed that the mineral-to-collagen ratio is higher in the middle to distal region of the cranial side of the non-loaded bones (*p* < 0.05, Fig. 1f). In contrast, the loaded bones exhibit similar degree of mineral to collagen along the middle of the bone, but lower at the most proximal and distal ends of the bones; these differences along the length show a trend but are not significant (*p* = 0.09) (Fig. 1f).

#### Bone material properties

The analysis of the unloading slope (RPI) revealed that for the cranial region, there is a significant difference along the length of the bone for both the non-loaded (+200% proximal vs. middle and distal; *p* < 0.001) and loaded (+50% proximal vs. middle, +50% distal vs. middle; *p* < 0.05) bones (Fig. 3c–f). Furthermore, a comparison of each position along the bone revealed that there was a significant difference between the non-loaded and loaded bones at the proximal end of the bones (+200% for the non-loaded; *p* < 0.01) and at the middle of the bones (+6% for the non-loaded; *p* < 0.05).

## Discussion

This study investigated the presence of microscopic heterogeneity in mouse bone with and without external loading. We also demonstrated local site-specific microscopic differences within bone tissue under normal ambulatory circumstances. In response to applied axial mechanical loading, the collagen content, mineral content, and mineral size as well as toughness are altered site-specifically. These data confirm our hypothesis that both structural and material properties vary across different anatomical regions of long bones not only on a macro- but also on a micro- and molecular scale and that these regions adapt site-specifically at each level in response to axial loading.

This study confirms earlier results that loading increases cortical BMD, area, and thickness [24, 25]. We site-specifically quantified the cortical thickness obtained during physiological loading and found that it is not consistent around the bone. The caudal site of cortical bone is thicker than any other region, indicating that a larger thickness is needed in order to sustain the load applied. In response to load, a thicker cortex is formed caudally, as shown, but not quantified, previously [17, 25]. We conclude that during habitual loading conditions, as well as during additional axial loading, cortical thickness is adjusted site-specifically in order to resist the load.

Using Raman spectroscopy, we could demonstrate, in non-loaded normally ambulatory tibiae, that the cranial site is more mineralized and the mineral crystals are smaller compared to those of the caudal site, indicating that the cranial site is stiffer. Taken together, this suggests that there are site-specific differences in bone material properties in the normal ambulatory tibiae. The differences between sites around the bone observed in the spectra emphasize the complex and localized nature of mineralization of the bone matrix. The material properties of bone, such as elastic modulus and toughness, are also affected by the degree of mineralization.

Using finite element analysis (FEM) of tibiae, previous studies have shown that in response to external axial loading, an intermediate tension is formed in the proximal/middle cranial side of tibiae, while high compressive strains are formed on the proximal/middle caudal side of tibiae [16, 36]. Mechanical laws relating material properties of the tissue to the stress and strain states imply that when tibiae are loaded axially, a certain force results in less strain in a higher mineralized region (i.e. within the cranial area experiencing tension), compared to a less mineralized region (the caudal area). Most theories suggest a relationship where increased strain results in increased bone formation. Thus, a greater stimulation of bone formation is expected in the less mineralized caudal compressive area (since lower mineralization results in higher strain levels), compared to the more mineralized tensile cranial area [16, 37]. Another explanation could be that the site-specific differences in strain and bone formation are due to the bone shape and cross section, as demonstrated in several papers using FEM [36, 38, 39]. Indeed, both theories suggest that more bone would form site-specifically in response to load-induced increase in strain, and results from this study confirm a significant in vivo, site-specific increase in cortical thickness due to loading in line with these predictions.

The results from this study show that regardless of whether the bone is loaded externally or not, there are differences in level of mineralization between the caudal and cranial sites, and they respond differently to load. New immature bone contains smaller mineral crystals, and smaller crystals are associated with reduced fracture risk [40]. Thus, it remains to be investigated whether the smaller crystals seen in response to load will remain small even when the bone matures. If so, load could result in smaller crystals, providing yet another mechanism whereby load would render bone more resistant to fracture at least initially. The mineralization along the length of the non-loaded bones is in agreement with a previous study, showing that the middle region of the tibiae is more mineralized than either end [20]. However, our loaded bones display an altered pattern of mineralization, where the material changes after loading are most enhanced at the proximal/middle and distal ends.

A limitation of this study is that we have used young adult mice and bone fragility disorders often affect elderly humans. However, a recent publication by Aido et al. show that loading led to enhanced collagen maturity and mineral/matrix ratio in the periosteum of the mid-shaft of elderly mouse bone, indicating that our results may hold true also for old bone [19].

Although the mice did not differ in age, gender, size, or weight, and the load was the same in all of the mice, the spread of the Raman data across the cranial and caudal regions, in both the non-loaded and loaded bones, is different from each other. This could be interpreted in several ways. First, this shows that there are inherent site-specific differences in the bone matrix chemistry across normal bone. Second, this confirms that the bones not only respond to load, but that they respond to load in different ways at different sites. Loaded bones are significantly more mineralized at the cranial tension sites than at the caudal compression sites. Third, this analysis highlights that the cranial sites contain bone with less mature mineral crystals both in the non-loaded and loaded bones compared to the caudal site. Fourth, finite element analysis has shown that the strains differ along the length of the bone when loaded axially [36].

Loading significantly decreased crystallinity, i.e. the crystals were less mature [28, 29], as measured by Raman spectroscopy both at the cranial and caudal sites. The decreased crystal size at the caudal region in response to load was confirmed by SAXS analysis showing a significant decrease in crystal plate thickness at the caudal region. [40]. However, SAXS analysis was performed on a transverse area containing both old and newly formed bone and could therefore only confirm the Raman analysis (as performed on the bone surface where new bone is expected) when a larger part of the analyzed bone area contained newly formed bone as in the caudal region. Another explanation to the difference in crystallinity could be that our loading procedure has caused micro-damage and subsequent intracortical remodeling. Although we cannot exclude the possibility that micro-damage could induce the differences seen in mineral composition, it has previously been shown that axial loading using the same protocol as used in this study with the same load did not induce micro-cracks [16]. It has also been shown that treadmill exercise significantly decreased the number of micro-cracks [41], indicating that intermediate loading would prevent development of micro-cracks. It is well known that the most load-sensitive region with regards to bone formation is the caudal and proximal bone from the knee to the mid shaft [24, 25, 36]. In our study, most changes in mineralization in response to load are seen at the cranial and proximal/middle as well as the distal regions of the tibiae, while most bone is formed caudally. Thus, the sites where most changes in mineralization are observed do not always correspond to the regions with highest bone formations in response to load. This indicates that the mineral content may not be the driving force for the bone anabolic response to loading. Instead, the driving force for bone formation could be strain [16, 36]. Within the normal range, the resistance to fracture is inversely related to the mineralization ratio, where tougher bone is more resistant to fracture. Owing to the natural variation along the length of the bones, the RPI data was very heterogeneous. A comparison of the bones at the proximal-middle region of the bones showed that the total indentation distance (TID) was lower, and the bone is therefore stiffer, at the cranial side in the loaded compared to non-loaded bones, further supporting the production of new, less mineralized bone, in response to load at the proximal site. However, this was not the case for the caudal side of the bone, which has a higher TID and would therefore be more susceptible to fracture [35]. The stiffness of the bones was not significantly different, although there was more variance in stiffness across the loaded bones on the cranial side. Upon loading, the stiffness of the bones changes mostly at the proximal end of the bones for the cranial site but more at the distal end for the caudal side.

A limitation of this study is that we investigated the bone response after 2 weeks of loading, in a dynamic time-period where we can expect new bone to be formed, but not yet reached a steady state. This enables us to study the process whereby new bone is being formed to adapt to the new imposed loading conditions, but we do not know if these differences will persist as the bone matures under the new loading conditions. In addition, we cannot exclude that the changes in bone quality observed could be due to differences in the proportion of new bone tissue compared to mature bone in the regions examined. However, both Raman spectroscopy and RPI analyses are performed at the bone surface where new bone has been repeatedly found after axial loading [16, 17, 25, 36], indicating that we are indeed mainly studying the quality of the newly formed bone. Previous studies have shown that short-term loading only alters the chemical properties of new periosteal and endosteal modeled bone, and not the old intracortical bone, in response to load [19, 42], further indicating that the changes in bone chemistry that we see in response to loading are mainly due to the newly formed bone and not alterations in the old intracortical bone.

Our study has several strengths. We used adult mice, which were thus not rapidly growing, in order to minimize the influence of growth. The axial loading protocol is well established with reproducible loading responses [17, 24–26]. We used two surface techniques, Raman and reference point indentation (RPI), to be able to analyze only the load-induced newly formed bone on the periosteum and not the old intracortical bone. The results of these techniques were supported with μCT and SAXS analysis that although not able to distinguish between new and old bone, they still confirmed the Raman and RPI analyses showing site-specific alterations in response to load.

Naturally, as in all animal studies, the obtained results may not be directly extrapolated to the human situation. The human tibia differs from the mouse tibia in shape. However, like mouse tibiae, human tibiae have curvatures and differ in cranial and caudal regions. As for mice, human bone size and shape differ in different regions of the bones [43]. There is also evidence that there is a broad heterogeneity of mineral to matrix across the human skeleton [10, 37, 44, 45], in line with our study in mice.

This new information also advances understanding by highlighting the importance of a site-specific response to load and providing more details on how the cranial and caudal sites differ on a microscopic level. The fact that bone is heterogeneous on a macroscopic level is not new, but the implications of inducing a change in heterogeneity also on the microscopic level through loading could be important. It is not yet investigated whether established bone anti-resorptive agents interfere, or not, with the possible site-specific heterogeneous adaptation to strain in human bone. It is important to understand the mechanisms underlying the site-specific heterogeneous response of bone sparing and bone anabolic drugs in human bone.

In conclusion, mouse bone material is site-specific, finely tuned, adaptive, and load responsive not only at the macro-scale (shape and size) but also at the micro-scale (collagen content and degree of mineralization). It would be beneficial if upcoming drugs to treat bone fragility disorders can take this mechanistic approach into account, so that new drugs do not interfere with the site-specific adaption to strain.

## Electronic supplementary material

Figure S1 Strain vs. Load curve Strain vs. load curve from ex vivo strain measurements displayed as mean ± SEM. (GIF 9 kb).

High resolution image (TIFF 72 kb).

Figure S2. Sites where measurements were performed Representative images of the sites for RPI, Raman, SAXS and μCT analysis are shown. (a) RPI measurements of the caudal and cranial sites of the bones were acquired from the crosshairs on the diagram. Raman spectra were acquired at the same sites and the additional blue stars. Dashed lines indicate the sites of analysis for (b) SAXS and (c) μCT analysis. (GIF 16 kb).

High resolution image (TIFF 129 kb).

## References

[1] Viguet-Carrin S, Garnero P, Delmas PD (2006). The role of collagen in bone strength. Osteoporos Int.

[2] Donnelly E (2011). Methods for assessing bone quality: a review. Clin Orthop Relat Res.

[3] Prince R, Draper C (2000). Bone and calcium.

[4] Baron R (2003) General principles of bone biology.

[5] Niyibizi C, Eyre DR (1989). Bone type V collagen: chain composition and location of a trypsin cleavage site. Connect Tissue Res.

[6] Bach-Gansmo FL, Weaver JC, Jensen MH, Leemreize H, Mader KS, Stampanoni M, Bruel A, Thomsen JS, Birkedal H (2015). Osteocyte lacunar properties in rat cortical bone: differences between lamellar and central bone. J Struct Biol.

[7] Shipov A, Zaslansky P, Riesemeier H, Segev G, Atkins A, Shahar R (2013). Unremodeled endochondral bone is a major architectural component of the cortical bone of the rat (*Rattus norvegicus*). J Struct Biol.

[8] Fratzl P, Roschger P, Fratzl-Zelman N, Paschalis EP, Phipps R, Klaushofer K (2007). Evidence that treatment with risedronate in women with postmenopausal osteoporosis affects bone mineralization and bone volume. Calcif Tissue Int.

[9] Turunen MJ, Kaspersen JD, Olsson U, Guizar-Sicairos M, Bech M, Schaff F, Tagil M, Jurvelin JS, Isaksson H (2016). Bone mineral crystal size and organization vary across mature rat bone cortex. J Struct Biol.

[10] Gourion-Arsiquaud S, Lukashova L, Power J, Loveridge N, Reeve J, Boskey AL (2013). Fourier transform infrared imaging of femoral neck bone: reduced heterogeneity of mineral-to-matrix and carbonate-to-phosphate and more variable crystallinity in treatment-naive fracture cases compared with fracture-free controls. J Bone Miner Res.

[11] Renders GA, Mulder L, van Ruijven LJ, Langenbach GE, van Eijden TM (2011). Mineral heterogeneity affects predictions of intratrabecular stress and strain. J Biomech.

[12] Boskey AL, Spevak L, Weinstein RS (2009). Spectroscopic markers of bone quality in alendronate-treated postmenopausal women. Osteoporos Int.

[13] Donnelly E, Meredith DS, Nguyen JT, Gladnick BP, Rebolledo BJ, Shaffer AD, Lorich DG, Lane JM, Boskey AL (2012). Reduced cortical bone compositional heterogeneity with bisphosphonate treatment in postmenopausal women with intertrochanteric and subtrochanteric fractures. J Bone Miner Res.

[14] Gourion-Arsiquaud S, Allen MR, Burr DB, Vashishth D, Tang SY, Boskey AL (2010). Bisphosphonate treatment modifies canine bone mineral and matrix properties and their heterogeneity. Bone.

[15] Sugiyama T, Meakin LB, Galea GL, Jackson BF, Lanyon LE, Ebetino FH, Russell RG, Price JS (2011). Risedronate does not reduce mechanical loading-related increases in cortical and trabecular bone mass in mice. Bone.

[16] De Souza RL, Matsuura M, Eckstein F, Rawlinson SC, Lanyon LE, Pitsillides AA (2005). Non-invasive axial loading of mouse tibiae increases cortical bone formation and modifies trabecular organization: a new model to study cortical and cancellous compartments in a single loaded element. Bone.

[17] Windahl SH, Saxon L, Borjesson AE (2013). Estrogen receptor-alpha is required for the osteogenic response to mechanical loading in a ligand-independent manner involving its activation function 1 but not 2. J Bone Miner Res.

[18] Isaksson H, Tolvanen V, Finnila MA (2009). Long-term voluntary exercise of male mice induces more beneficial effects on cancellous and cortical bone than on the collagenous matrix. Exp Gerontol.

[19] Aido M, Kerschnitzki M, Hoerth R, Checa S, Spevak L, Boskey AL, Fratzl P, Duda GN, Wagermaier W, Willie BM (2015). Effect of in vivo loading on bone composition varies with animal age. Exp Gerontol.

[20] Buckley K, Kerns JG, Birch HL, Gikas PD, Parker AW, Matousek P, Goodship AE (2014). Functional adaptation of long bone extremities involves the localized “tuning” of the cortical bone composition; evidence from Raman spectroscopy. J Biomed Opt.

[21] Isaksson H, Turunen MJ, Rieppo L, Saarakkala S, Tamminen IS, Rieppo J, Kroger H, Jurvelin JS (2010). Infrared spectroscopy indicates altered bone turnover and remodeling activity in renal osteodystrophy. J Bone Miner Res.

[22] Matousek P, Morris MD, Everall N, Clark IP, Towrie M, Draper E, Goodship A, Parker AW (2005). Numerical simulations of subsurface probing in diffusely scattering media using spatially offset Raman spectroscopy. Appl Spectrosc.

[23] Buckley K, Matousek P, Parker A, Goodship A (2012). Raman spectroscopy reveals differences in collagen secondary structure which relate to the levels of mineralisation in bones that have evolved for different functions. J Raman Spectrocopy.

[24] Galea GL, Hannuna S, Meakin LB, Delisser PJ, Lanyon LE, Price JS (2015). Quantification of alterations in cortical bone geometry using site specificity software in mouse models of aging and the responses to ovariectomy and altered loading. Front Endocrinol (Lausanne).

[25] Sugiyama T, Price JS, Lanyon LE (2010). Functional adaptation to mechanical loading in both cortical and cancellous bone is controlled locally and is confined to the loaded bones. Bone.

[26] Todd H, Galea GL, Meakin LB, Delisser PJ, Lanyon LE, Windahl SH, Price JS (2015). Wnt16 is associated with age-related bone loss and estrogen withdrawal in murine bone. PLoS One.

[27] Karampas IA, Orkoula MG, Kontoyannis CG (2013). A quantitative bioapatite/collagen calibration method using Raman spectroscopy of bone. J Biophotonics.

[28] Goodyear SR, Gibson IR, Skakle JM, Wells RP, Aspden RM (2009). A comparison of cortical and trabecular bone from C57 Black 6 mice using Raman spectroscopy. Bone.

[29] McElderry JD, Zhu P, Mroue KH et al (2013) Crystallinity and compositional changes in carbonated apatites: evidence from 31P solid-state NMR, Raman, and AFM analysis. J Solid State Chem 206. doi:10.1016/j.jssc.2013.08.011 10.1016/j.jssc.2013.08.011 PMC3835554 24273344

[30] Turunen MJ, Lages S, Labrador A, Olsson U, Tagil M, Jurvelin JS, Isaksson H (2014). Evaluation of composition and mineral structure of callus tissue in rat femoral fracture. J Biomed Opt.

[31] Bunger MH, Oxlund H, Hansen TK, Sorensen S, Bibby BM, Thomsen JS, Langdahl BL, Besenbacher F, Pedersen JS, Birkedal H (2010). Strontium and bone nanostructure in normal and ovariectomized rats investigated by scanning small-angle X-ray scattering. Calcif Tissue Int.

[32] Fratzl P, Schreiber S, Klaushofer K (1996). Bone mineralization as studied by small-angle x-ray scattering. Connect Tissue Res.

[33] Rinnerthaler S, Roschger P, Jakob HF, Nader A, Klaushofer K, Fratzl P (1999). Scanning small angle X-ray scattering analysis of human bone sections. Calcif Tissue Int.

[34] Kaspersen JD, Turunen MJ, Mathavan N, Lages S, Pedersen JS, Olsson U, Isaksson H (2016). Small-angle X-ray scattering demonstrates similar nanostructure in cortical bone from young adult animals of different species. Calcif Tissue Int.

[35] Diez-Perez A, Guerri R, Nogues X (2010). Microindentation for in vivo measurement of bone tissue mechanical properties in humans. J Bone Miner Res.

[36] Moustafa A, Sugiyama T, Prasad J, Zaman G, Gross TS, Lanyon LE, Price JS (2012). Mechanical loading-related changes in osteocyte sclerostin expression in mice are more closely associated with the subsequent osteogenic response than the peak strains engendered. Osteoporos Int.

[37] Sugiyama T, Meakin LB, Browne WJ, Galea GL, Price JS, Lanyon LE (2012). Bones' adaptive response to mechanical loading is essentially linear between the low strains associated with disuse and the high strains associated with the lamellar/woven bone transition. J Bone Miner Res.

[38] Patel TK, Brodt MD, Silva MJ (2014). Experimental and finite element analysis of strains induced by axial tibial compression in young-adult and old female C57Bl/6 mice. J Biomech.

[39] Willie BM, Birkhold AI, Razi H, Thiele T, Aido M, Kruck B, Schill A, Checa S, Main RP, Duda GN (2013). Diminished response to in vivo mechanical loading in trabecular and not cortical bone in adulthood of female C57Bl/6 mice coincides with a reduction in deformation to load. Bone.

[40] Chatterji S, Wall JC, Jeffery JW (1981). Age-related changes in the orientation and particle size of the mineral phase in human femoral cortical bone. Calcif Tissue Int.

[41] Kohn DH, Sahar ND, Wallace JM, Golcuk K, Morris MD (2009). Exercise alters mineral and matrix composition in the absence of adding new bone. Cells Tissues Organs.

[42] Checa S, Hesse B, Roschger P, Aido M, Duda GN, Raum K, Willie BM (2015). Skeletal maturation substantially affects elastic tissue properties in the endosteal and periosteal regions of loaded mice tibiae. Acta Biomater.

[43] Wang Q, Seeman E (2013) Skeletal growth and peak bone strength 10.1016/j.beem.2008.07.008 19028352

[44] Zimmermann EA, Schaible E, Bale H, Barth HD, Tang SY, Reichert P, Busse B, Alliston T, Ager JW, Ritchie RO (2011). Age-related changes in the plasticity and toughness of human cortical bone at multiple length scales. Proc Natl Acad Sci U S A.

[45] Turunen MJ, Prantner V, Jurvelin JS, Kroger H, Isaksson H (2013). Composition and microarchitecture of human trabecular bone change with age and differ between anatomical locations. Bone.

