# Correction to: Compressive loading of the murine tibia reveals site-specific micro-scale differences in adaptation and maturation rates of bone

**DOI:** 10.1007/s00198-018-4496-7

**Published:** 2018-07-09

**Authors:** I. Bergström, J. G. Kerns, A. E. Törnqvist, C. Perdikouri, N. Mathavan, A. Koskela, H. B. Henriksson, J. Tuukkanen, G. Andersson, H. Isaksson, A. E. Goodship, S. H. Windahl

**Affiliations:** 1 Department of Endocrinology, Metabolism and Diabetes, Karolinska University Hospital, Karolinska Institutet, Stockholm, Sweden; 2 UCL Institute of Orthopedics and Musculoskeletal Science, Royal National Orthopedic Hospital, London, UK; 3 Lancaster Medical School, Faculty of Health and Medicine, Lancaster University, Lancaster, LA1 4YG UK; 4 Rheumatology and Bone Diseases Unit, Centre for Genomic and Experimental Medicine, MRC Institute of Genetics and Molecular Medicine, Western General Hospital, University of Edinburgh, Edinburgh, EH4 2XU UK; 5 Department of Biomedical Engineering and Department of Orthopedics, Lund University, Lund, Sweden; 6 Institute of Cancer and Translational Medicine, Department of Anatomy and Cell Biology, MRC Oulu, University of Oulu, Oulu, Finland; 7 Department of Orthopedics, Institute of Clinical Sciences, Sahlgrenska Academy, University of Gothenburg, Gothenburg, Sweden; 8 Department of Orthopedics, Sahlgrenska University Hospital, Gothenburg, Sweden; 9 Department of Laboratory Medicine, Division of Pathology, Karolinska University Hospital, Karolinska Institutet, Huddinge, Stockholm, Sweden; 10 Centre for Comparative and Clinical Anatomy, School of Veterinary Science, University of Bristol, Bristol, UK; 11 Centre for Bone and Arthritis Research, Institute of Medicine, Sahlgrenska Academy, University of Gothenburg, Gothenburg, Sweden; 12 Present Address: Department of Laboratory Medicine, Division of Pathology, Karolinska Institutet F46, Karolinska University Hospital, Huddinge, 141 86 Sweden

**Correction to: Osteoporos Int (2017) 28:1121–1131**

10.1007/s00198-016-3846-6

This article was originally published under a CC BY-NC-ND 4.0 license, but has now been made available under a CC BY 4.0 license. The PDF and HTML versions of the paper have been modified accordingly.

