# Supplementary figures and images for: Compressive loading of the murine tibia reveals site-specific micro-scale differences in adaptation and maturation rates of bone
Source: Osteoporos Int. 2016 Dec 5;28(3):1121–31. doi: 10.1007/s00198-016-3846-6 (PMC5306148; doi:10.1007/s00198-016-3846-6)

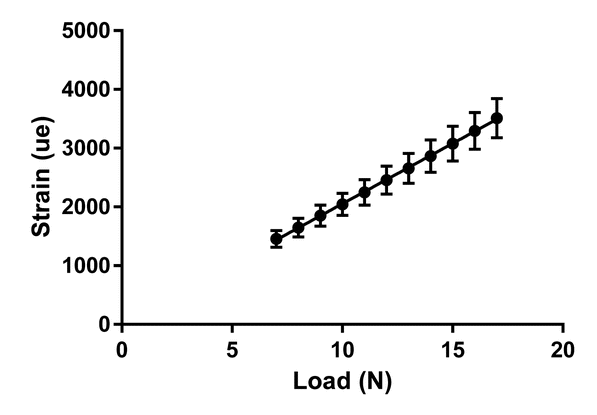

Supplement: Supplementary file 1 — Strain vs. Load curve Strain vs. load curve from ex vivo strain measurements displayed as mean ± SEM. (GIF 9 kb) [file 198_2016_3846_Fig1_ESM.gif]

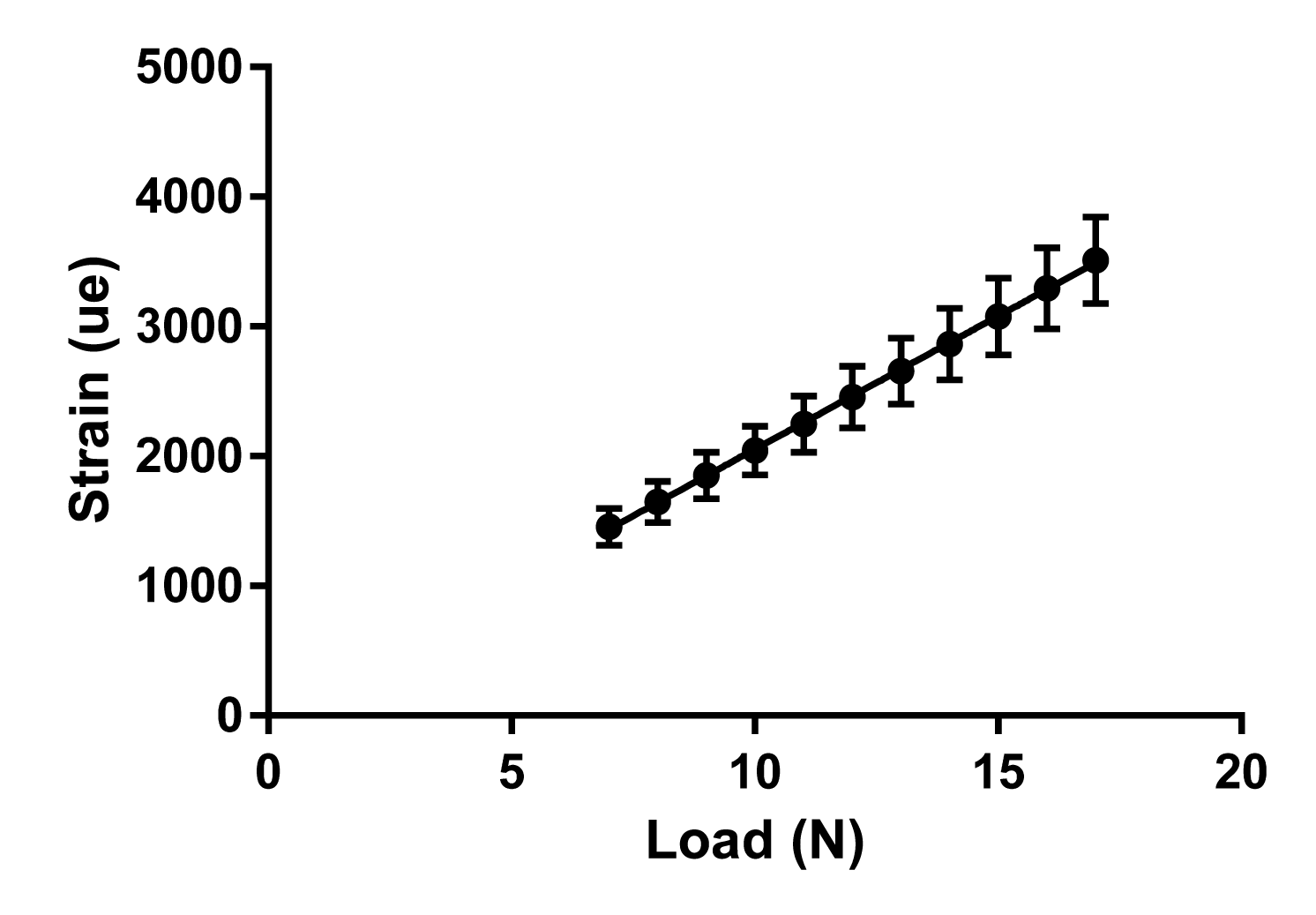

Supplement: Supplementary file 2 — High resolution image (TIFF 72 kb) [file 198_2016_3846_MOESM1_ESM.tif]

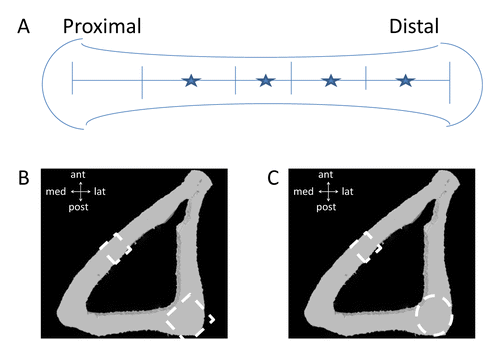

Supplement: Supplementary file 3 — Sites where measurements were performed Representative images of the sites for RPI, Raman, SAXS and μCT analysis are shown. (a) RPI measurements of the caudal and cranial sites of the bones were acquired from the crosshairs on the diagram. Raman spectra were acquired at the same sites and the additional blue stars. Dashed lines indicate the sites of analysis for (b) SAXS and (c) μCT analysis. (GIF 16 kb) [file 198_2016_3846_Fig2_ESM.gif]

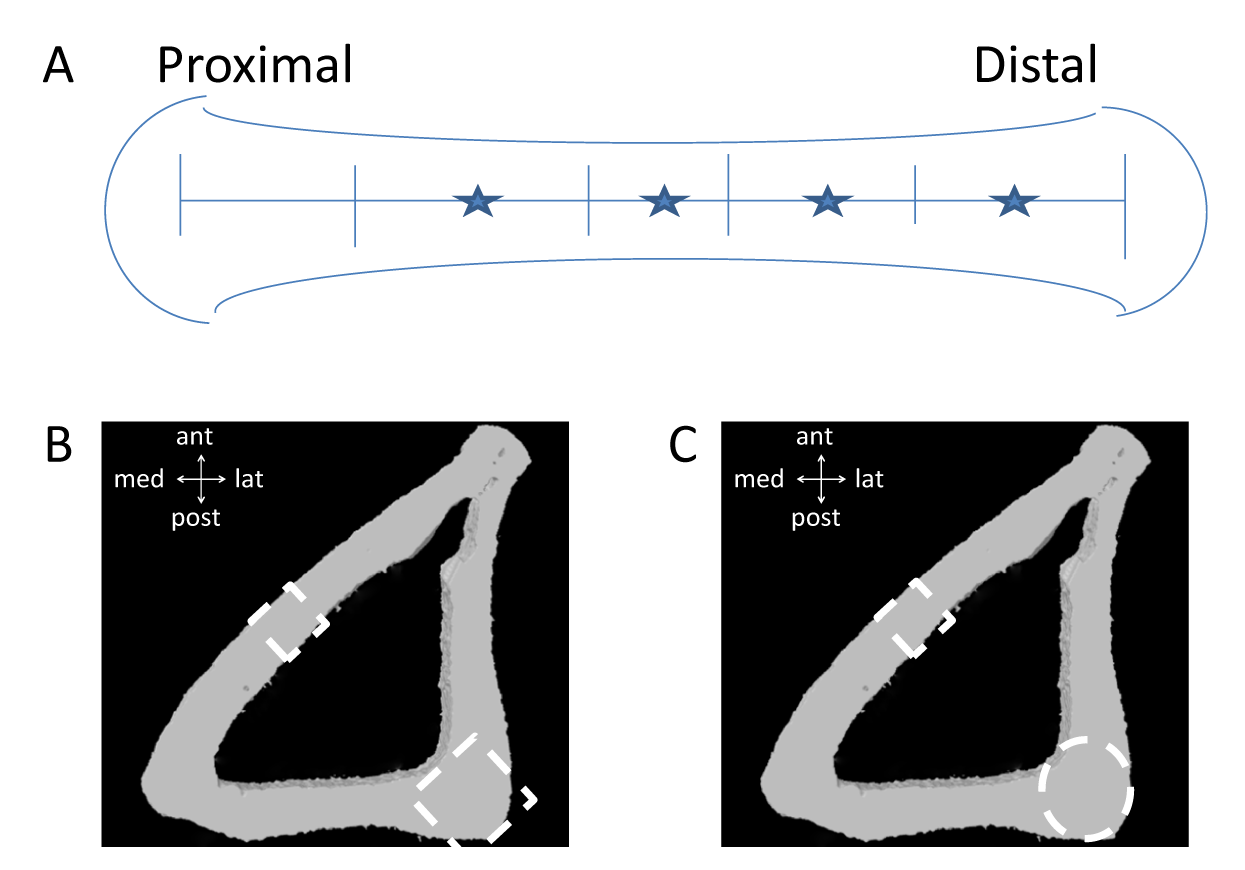

Supplement: Supplementary file 4 — High resolution image (TIFF 129 kb) [file 198_2016_3846_MOESM2_ESM.tif]
